# Hsp90 Is Involved in Apoptosis of *Candida albicans* by Regulating the Calcineurin-Caspase Apoptotic Pathway

**DOI:** 10.1371/journal.pone.0045109

**Published:** 2012-09-18

**Authors:** BaoDi Dai, Yan Wang, DeDong Li, Yi Xu, RongMei Liang, LanXue Zhao, YongBing Cao, JianHui Jia, YuanYing Jiang

**Affiliations:** 1 School of Pharmacy, Second Military Medical University, Shanghai, China; 2 Department of Pharmacy, General Hospital of Jinan Military Command Region, Jinan, China; 3 Department of Clinical Pharmacy, General Hospital of Chengdu Military Command Region, Chengdu, China; 4 Department of Pharmacology, School of Life Science and Biopharmacology, Shenyang Pharmaceutical University, Shenyang, China; University of Minnesota, United States of America

## Abstract

*Candida albicans* is the most common human fungal pathogen. Recent evidence has revealed the occurrence of apoptosis in *C. albicans* that is inducible by environmental stresses such as hydrogen peroxide, acetic acid, and amphotericin B. Apoptosis is regulated by the calcineurin-caspase pathway in *C. albicans*, and calcineurin is under the control of Hsp90 in echinocandin resistance. However, the role of Hsp90 in apoptosis of *C. albicans* remains unclear. In this study, we investigated the role of Hsp90 in apoptosis of *C. albicans* by using an Hsp90-compromised strain tetO-HSP90/hsp90 and found that upon apoptotic stimuli, including hydrogen peroxide, acetic acid or amphotericin B treatment, less apoptosis occurred, less ROS was produced, and more cells survived in the Hsp90-compromised strain compared with the Hsp90/Hsp90 wild-type strain. In addition, Hsp90-compromised cells were defective in up-regulating caspase*-*encoding gene *CaMCA1* expression and activating caspase activity upon the apoptotic stimuli. Investigations on the relationship between Hsp90 and calcineurin revealed that activation of calcineurin could up-regulate apoptosis but could not further down-regulate apoptosis in Hsp90-compromised cells, indicating that calcineurin was downstream of Hsp90. Hsp90 inhibitor geldanamycin (GdA) could further decrease the apoptosis in calcineurin-pathway-defect strains, indicating that compromising Hsp90 function had a stronger effect than compromising calcineurin function on apoptosis. Collectively, this study demonstrated that compromised Hsp90 reduced apoptosis in *C. albicans*, partially through downregulating the calcineurin-caspase pathway.

## Introduction

*Candida albicans* is the leading fungal pathogen and may cause a variety of infections in immunocompromised individuals. The frequency of fungal infections continues to increase in concert with the growing immunocompromised patient population, including individuals infected with HIV and those undergoing chemotherapy, major surgery, or solid organ transplantation [1]–[3]. In addition, the many antifungal drugs in clinical use target ergosterol or its biosynthesis due to the limited number of drug targets available to exploit in fungal pathogens that are absent or sufficiently divergent in human hosts. Thus, many current antifungal therapies have unfortunate clinical side effects. Besides, more clinical isolates are resistant to traditional antifungal drugs [4]. Therefore it is necessary to develop new antifungal strategies. Uncovering the mechanistic basis of cell death decisions in fungi may well provide new development in the search for novel antifungal agents.

Recent evidence has revealed the occurrence of apoptosis in *C. albicans* which can be induced by various environmental stimuli such as hydrogen peroxide (H_2_O_2_), acetic acid (AA) and amphotericin B (AMB) [5]–[8]. *C. albicans* cells undergo a series of physiological changes during apoptosis, including chromatin condensation, nuclear fragmentation and increased production of reactive oxygen species (ROS). Now, ROS production is regarded as one of the typical hallmarks of apoptosis [7]–[9].

Apoptosis is a complex process involving multiple factors [9]–[16], including *YCA1* [9] and calcineurin [14]. *YCA1* encodes a metacaspase in *Saccharomyces cerevisiae*, which is functionally similar to mammalian caspase [9], [11], [13]. Upon apoptotic stimulation, metacaspase is activated, leading to the occurrence of apoptosis. We have reported previously that H_2_O_2_-induced apoptosis in *C. albicans* was accompanied by activation of *CaMCA1*, the ortholog of *YCA1* [13]. Calcineurin, an important protein in Ca^2+^-dependent signal transduction pathway [15]–[17], is also related to apoptosis [14], [18], [19]. It consists of a catalytic subunit A (encoded by *CNA1*) and a regulatory subunit B (encoded by *CNB1*) [20], and acts on transcriptional factor Crz1p [21]–[23]. Its function can be compromised by Cyclosporin A pharmacologically. Recently, we reported that calcineurin and Crz1p are also required for H_2_O_2_-induced apoptosis in *C. albicans* through regulating *CaMCA1* expression and caspase activity [14].

**Figure 1:**
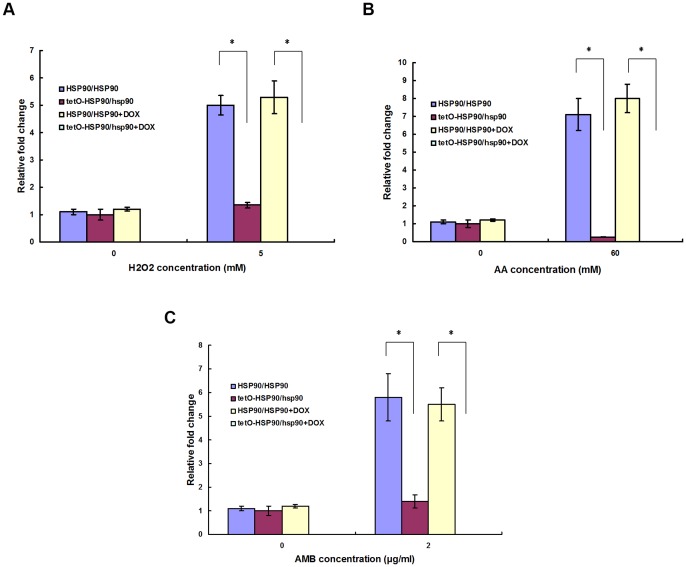
*HSP90* expression after apoptotic stimulus treatment. *C. albicans* cells were treated with or without apoptotic stimuli for 3 h in the absence or presence of 20 μg/ml DOX. (A) Cells treated with H_2_O_2_. (B) Cells treated with AA. (C) Cells treated with AMB. Transcriptional level of *HSP90* was detected through real-time RT-PCR and normalized on the basis of the 18S level. Data are shown as mean ± SD from three independent experiments.

Hsp90 is a crucial molecular chaperone in stress response of *C. albicans* [24]–[29] and has been reported to orchestrate echinocandin resistance via regulating calcineurin pathway [30]. Calcineurin pathway is involved in apoptosis [14], [18], [19], while the role of Hsp90 in apoptosis remains unclear. In this study, we investigated the role of Hsp90 in apoptosis of *C. albicans* and revealed that compromised Hsp90 attenuated apoptosis through regulating calcineurin pathway and caspase activity.

**Figure 2:**
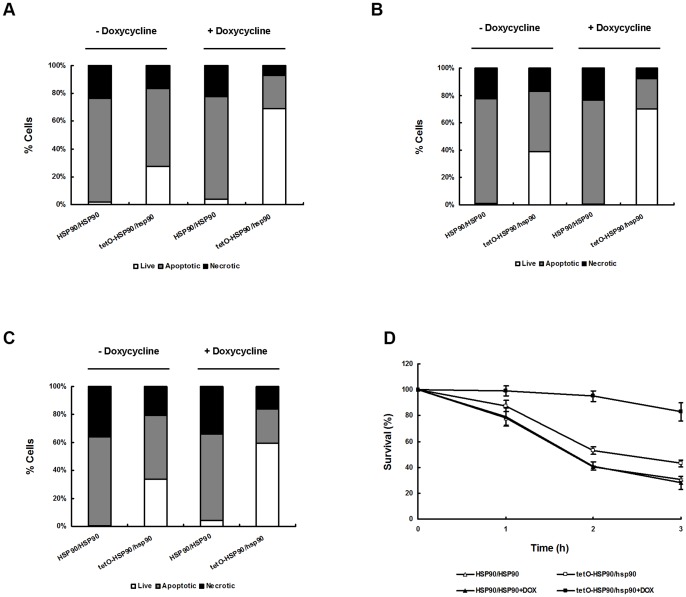
The impact of compromised Hsp90 on the fate of *C. albicans* cells after apoptotic stimuli. The number of live cells (white bars), apoptotic cells (grey bars) and necrotic cells (black bars) were assessed after apoptotic stimulus treatment for 3 h in the absence or presence of 20 μg/ml DOX. (A) Cells treated with 5 mM H_2_O_2_. (B) Cells treated with 60 mM AA. (C) Cells treated with 2 μg/ml AMB. (D) After 1.25 mM H_2_O_2_ treatment for 3 h, overall viability was determined by clonogentic assays. Data are shown as mean ± SD from three independent experiments.

## Results

### Compromised Hsp90 reduces apoptosis upon apoptotic stimulation

To investigate the role of Hsp90 in apoptosis of *C. albicans,* we compared the difference in apoptosis between wild-type HSP90/HSP90 strain and Hsp90-compromised strain upon three apoptotic stimuli. The Hsp90-compromised strain used in this study was an engineered strain tetO-HSP90/hsp90 with one copy of *HSP90* knocked out, while the other copy was inserted under a tetracycline promoter Tet-off [30]. In the tetO-HSP90/hsp90 strain, the expression of tetO-*HSP90* was inhibited in the presence of tetracycline or analog doxycycline (DOX), while it was induced when they were absent. Transcriptional repression of *HSP90* from tetO promoter in apoptosis was confirmed by real-time PCR detection (Fig. 1). After treatment with 5 mM H_2_O_2_ for 3 h, a 5-fold increase in *HSP90* expression was observed in wild-type strain. However, no change in *HSP90* expression was observed in the Hsp90-compromised tetO-HSP90/hsp90 strain in the presence of 20 μg/ml DOX (Fig. 1A). Similar results were also obtained from other apoptotic stimulations including AA or AMB treatment. After exposure to 60 mM AA for 3 h, a 7-fold increase in *HSP90* expression was observed in wild-type strain. In contrast, no change in *HSP90* expression was seen in tetO-HSP90/hsp90 strain in the presence of 20 μg/ml DOX (Fig. 1B). After treatment with AMB, a 5.8-fold increase in *HSP90* expression was observed in wild-type strain, while no change in *HSP90* expression was shown in tetO-HSP90/hsp90 strain in the presence of 20 μg/ml DOX (Fig. 1C).

**Table 1:** The percentage of cells in *C. albicans* after H_2_O_2_ treatment with or without DOX^1^.

Strains	Cells (%)		
	Live	Apoptotic	Necrotic
HSP90/HSP90	1.88±0.01	74.31±0.71	23.81±2.57
tetO-HSP90/hsp90	27.26±0.02^*^	56.13±0.51^*^	16.61±1.13
HSP90/HSP90+ DOX	3.69±0.33	74.10±1.34	22.21±1.92
tetO-HSP90/hsp90+DOX	69.12±5.13^*^	23.64±0.74^*^	7.24±0.97^*^

1 Three independent experiments were carried out and the results were presented as the mean ± SD. * indicated p<0.05 compared with the wild-type strain in the same DOX concentration.

**Table 2:** The percentage of cells in *C. albicans* after AA treatment with or without DOX^2^.

Strains	Cells (%)		
	Live	Apoptotic	Necrotic
HSP90/HSP90	0.73±0.01	77.00±2.01	22.27±1.88
tetO-HSP90/hsp90	39.01±1.45^*^	44.00±0.76^*^	16.99±1.58
	0.44±0.01	76.02±1.09	23.54±0.97
tetO-HSP90/hsp90+DOX	70.21±2.33^*^	22.01±0.04^*^	7.78±0.21^*^

2 Three independent experiments were carried out and the results were presented as the mean ± SD. * indicated p<0.05 compared with the wild-type strain in the same DOX concentration.

Given the different expressions of *HSP90*, we further investigated the impact of the apoptotic stimuli on the fate of the two strain cells using TUNEL assay to determine apoptotic cells and PI uptake assay to determine necrotic cells. After H_2_O_2_ treatment, a smaller proportion of cells were apoptotic or necrotic in the Hsp90-compromised tetO-HSP90/hsp90 strain compared to the wild-type strain, as shown in Fig. 2A and Table 1. Similar results were obtained from other apoptotic stimulations including AA (Fig. 2B and Table 2) or AMB (Fig. 2C and Table 3) treatment. These data indicated that compromised Hsp90 decreased apoptosis in *C. albicans*.

**Table 3:** The percentage of cells in *C. albicans* after AMB treatment with or without DOX^3^.

Strains	Cells (%)		
	Live	Apoptotic	Necrotic
HSP90/HSP90	0.47±0.01	63.58±0.77	35.95±1.66
tetO-HSP90/hsp90	33.67±3.11^*^	45.44±0.94^* `^	20.89±0.46^*^
HSP90/HSP90+ DOX	4.01±0.37	61.99±0.31	34.00±0.11
tetO-HSP90/hsp90+DOX	59.52±0.37^*^	24.88±0.23^*^	15.60±0.97^*^

3 Three independent experiments were carried out and the results were presented as the mean ± SD. * indicated p<0.05 compared with the wild-type strain in the same DOX concentration.

The overall viability experiment was carried out to analyze the fate of fungal cells after apoptotic stimulation. After treatment with H_2_O_2_, tetO-HSP90/hsp90 cells survived longer than wild-type cells (Fig. 2D). After exposure to 1.25 mM H_2_O_2_ for 3 h, a higher survival percentage was observed in tetO-HSP90/hsp90 cells as compared with wild-type strain cells. These data indicated that tetO-HSP90/hsp90 cells had more probability to survive upon apoptotic stimulation, which is consistent with the results of TUNEL and PI uptake assays.

**Figure 3:**
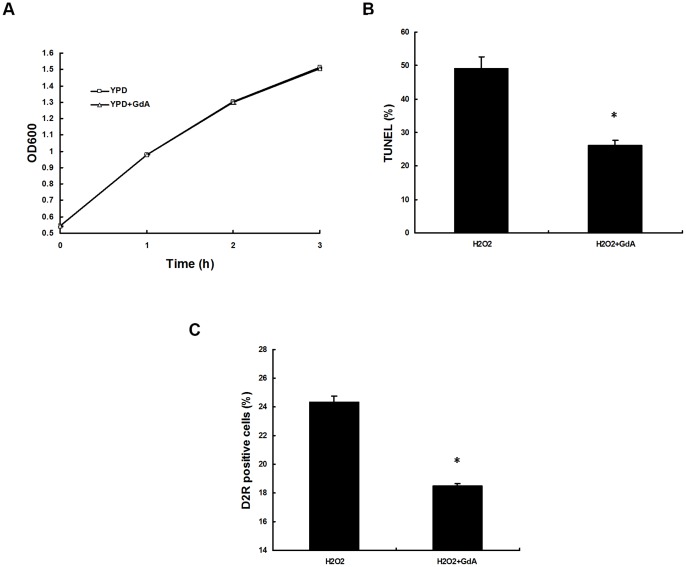
Pharmacological inhibition of Hsp90 with GdA decreased apoptosis and caspase activity of *C. albicans.* Wild-type HSP90/HSP90 strain cells were exposed to 1.25 mM H_2_O_2_ for 3 h in the absence or presence of GdA (0.5 μM). (A) The effect of 0.5 μM GdA on growth by OD_600_. *C. albicans* cells were grown in YPD to exponential phase (OD_600_ = 0.5) and then treated with or without 0.5 μM GdA. OD_600_ was detected at each time point. (B) Apoptotic cells were classified by TUNEL using flow cytometry. (C) Caspase activity was determined by D_2_R dye by flow cytometry. Results are shown as mean ± SD from three independent experiments. * indicates p<0.05 compared to the wild-type cells.

The role of Hsp90 in apoptosis was further investigated using an Hsp90 inhibitor, geldanamycin (GdA) (Fig. 3). The GdA concentration used in the experiment did not affect the growth of the normal strain without addition of the apoptotic stimuli, and there was no significant difference in OD_600_ between the blank control group and GdA group (Fig. 3A). However, the addition of GdA significantly decreased the proportion of apoptotic cells after 1.25 mM H_2_O_2_ treatment for 3 h (P<0.05, Fig. 3B), which is consistent with the results obtained from genetic depletion of *HSP90*.

**Figure 4:**
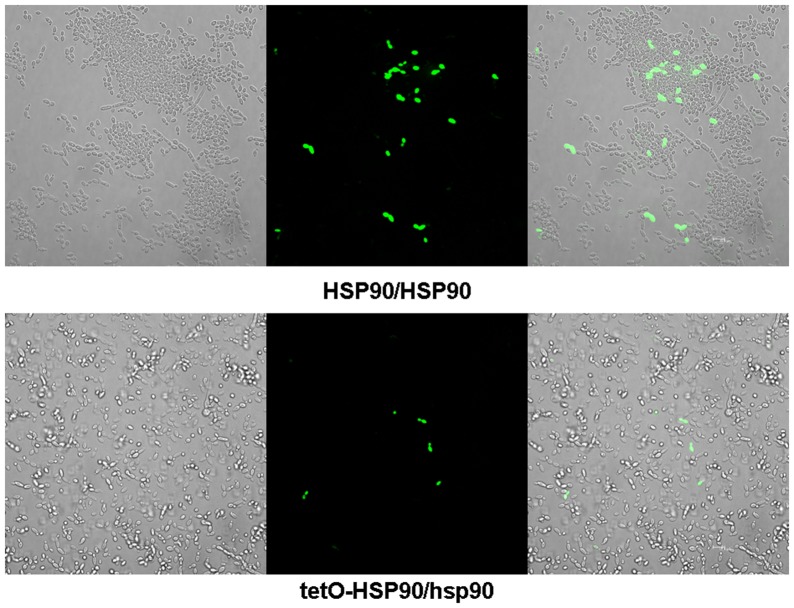
Representative confocal scanning laser fluorescence images of HSP90/HSP90 and tetO-HSP90/hsp90 cells stained for ROS accumulation following exposure to 5 mM H_2_O_2_ for 3 h in the presence of DOX. Bar represents 10.8 μm.

### Compromised Hsp90 reduces ROS production upon apoptotic stimulation

**Figure 5:**
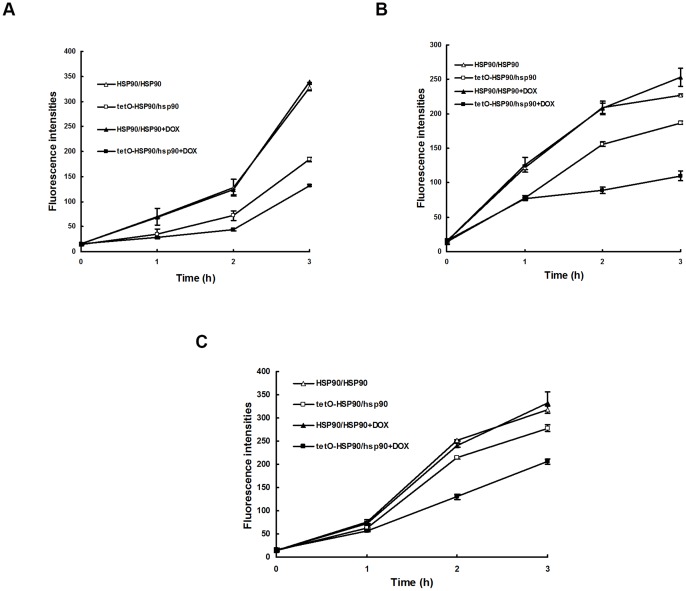
Intracellular ROS production after apoptotic stimuli. Cells were exposed to apoptotic stimuli for 3 h in the absence or presence of 20 μg/ml DOX, and intracellular ROS was detected by DCFH-DA. (A) Quantitative assay of ROS generation after 5 mM H_2_O_2_ treatment. (B) Quantitative assay of ROS generation after 60 mM AA treatment. (C) Quantitative assay of ROS generation after 2 μg/ml AMB treatment. Data shown are mean ± SD from three independent experiments.

We further investigated the impact of Hsp90 compromise on ROS production, knowing that ROS is regarded as one of the typical hallmarks of apoptosis. The results indicated that compromised Hsp90 reduced ROS production upon various apoptotic stimulations. The result of confocal laser microscopy showed that the fluorescence signal indicating intracellular ROS was obviously strong in HSP90/HSP90 cells after 5 mM H_2_O_2_ treatment for 3 h, while it was relatively weak in *HSP90* depleted tetO-HSP90/hsp90 cells in the presence of 20 μg/ml DOX (Fig. 4). This result was also confirmed by flow cytometry. When genetic depletion of *HSP90* was done by adding doxycycline, intracellular ROS in H_2_O_2_-treated tetO-HSP90/hsp90 cells decreased to about 50% of that in HSP90/HSP90 cells (Fig. 5A). Similar results were obtained by using 60 mM AA (Fig. 5B) or 2 μg/ml AMB (Fig. 5C) as the apoptotic stimuli. So after genetic depletion of *HSP90*, intracellular ROS production was seen to significantly decrease in tetO-HSP90/hsp90 cells compared with that in wild-type cells upon the same apoptotic stimulation, which is consistent with the results shown above.

### Compromised Hsp90 leads to defects in caspase activation upon apoptotic stimuli

To determine whether Hsp90 contributed to apoptosis through regulating *CaMCA1*/caspase pathway, we investigated the impact of Hsp90 on H_2_O_2_-induced *CaMCA1* expression and caspase activity by using real-time PCR and confocal laser microscopy.

**Figure 6:**
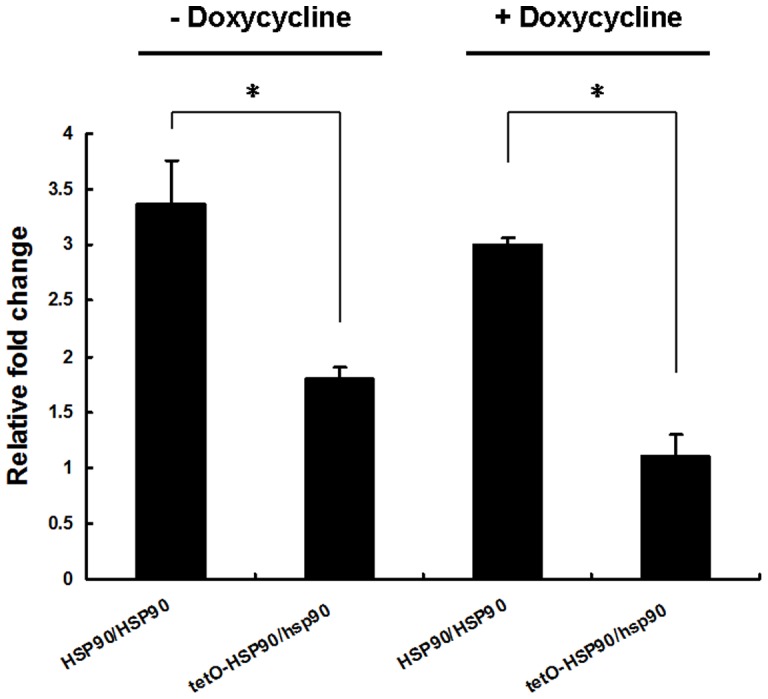
*CaMCA1* expression after H_2_O_2_ treatment. *C. albicans* cells were treated with 5 mM H_2_O_2_ for 3 h in the absence or presence of 20 μg/ml DOX. Transcriptional level of *CaMCA1* was detected by real-time RT-PCR and normalized on the basis of the 18S level. Data are shown as mean ± SD from three independent experiments. * indicates p<0.05 compared to the wild-type cells.

After 5 mM H_2_O_2_ treatment for 3 h, a 3-fold increase in *CaMCA1* expression was observed in the wild-type strain, while, only a 1.1-fold increase was observed in tetO-HSP90/hsp90 strain after compromising *HSP90* expression (Fig. 6), indicating that the Hsp90-compromised strain was defected and unable to activate *CaMCA1* expression upon H_2_O_2_ stimulus.

**Figure 7:**
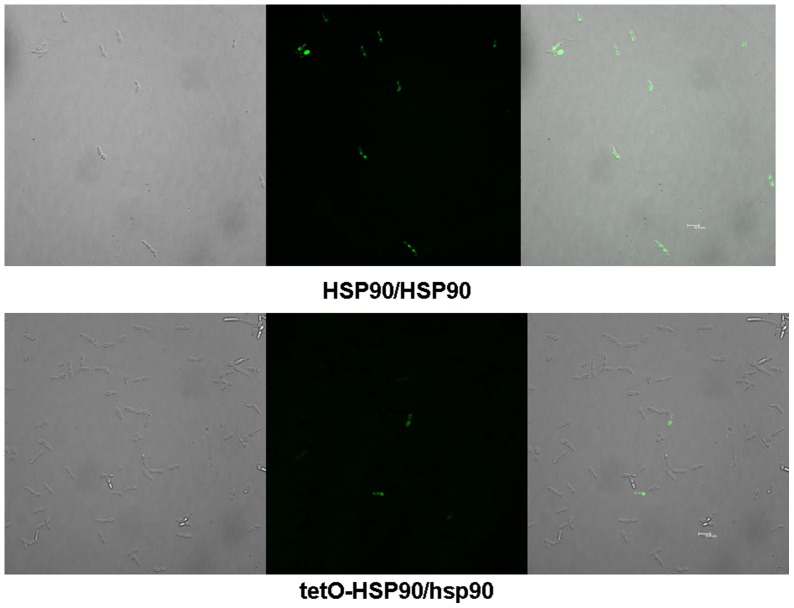
Representative confocal scanning laser fluorescence images of HSP90/HSP90 and tetO-HSP90/hsp90 cells stained for caspase activity following exposure to 5 mM H_2_O_2_ for 3 h in the presence of DOX. Bar represents 10.8 μm.

**Table 4:** Caspase activity of *C. albicans* cells after H_2_O_2_ treatment^4^.

Strains	5 mM H_2_O_2_
HSP90/HSP90	72.81±1.32
tetO-HSP90/hsp90	57.92±1.37^*^
HSP90/HSP90+DOX	71.99±0.48
tetO-HSP90/hsp90+DOX	35.42±1.49^*^

4 After treatment with or without 20 μg/ml DOX for 3 h, caspase activity of *C. albicans* cells was detected flow cytometry after D_2_R staining. Three independent experiments were carried out and the results were presented as the mean ± SD of D_2_R positive cells percentage. * indicated p<0.05 compared with the wild-type strain group.

**Table 5:** Caspase activity of *C. albicans* cells after AA treatment^5^.

Strains	60 mM AA
HSP90/HSP90	43.61±0.40
tetO-HSP90/hsp90	27.90±1.51^*^
HSP90/HSP90+DOX	42.43±3.38
tetO-HSP90/hsp90+DOX	18.59±0.16^*^

5 After treatment with or without 20 μg/ml DOX for 3 h, caspase activity of *C. albicans* cells was detected flow cytometry after D_2_R staining. Three independent experiments were carried out and the results were presented as the mean ± SD of D_2_R positive cells percentage. * indicated p<0.05 compared with the wild-type strain group.

**Table 6:** Caspase activity of *C. albicans* cells after AMB treatment^6^.

Strains	2 μg/ml AMB
HSP90/HSP90	50.72±0.49
tetO-HSP90/hsp90	32.22±0.45^*^
HSP90/HSP90+DOX	51.92±2.34
tetO-HSP90/hsp90+DOX	22.83±0.46^*^

6 After treatment with or without 20 μg/ml DOX for 3 h, caspase activity of *C. albicans* cells was detected flow cytometry after D_2_R staining. Three independent experiments were carried out and the results were presented as the mean ± SD of D_2_R positive cells percentage. * indicated p<0.05 compared with the wild-type strain group.

**Figure 8:**
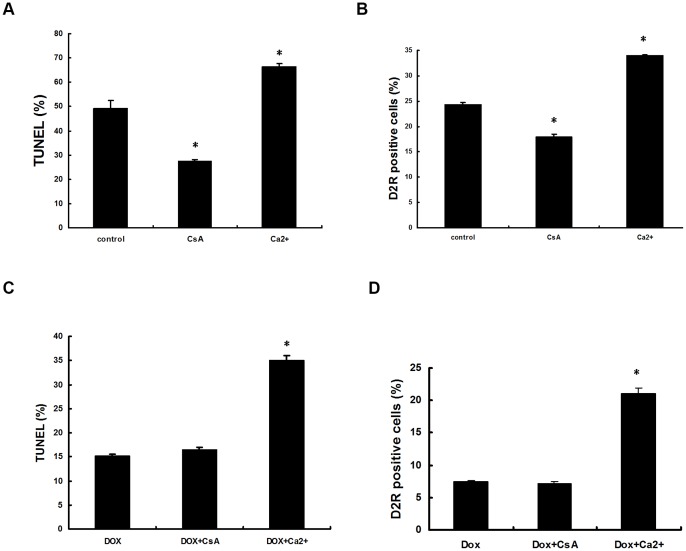
Calcineurin is downstream of Hsp90 in apoptosis. *C. albicans* cells were exposed to 1.25 mM H_2_O_2_ for 3 h in the absence or presence of CaCl_2_ (1 mM) or CysA (0.08 μM). (A) and (B) are wild-type HSP90/HSP90 strain cells. (C) and (D) are tetO-HSP90/hsp90 cells in the presence of 20 μg/ml DOX. Results are shown as mean ± SD from three independent experiments. * indicates p<0.05 compared to the wild-type cells.

**Figure 9:**
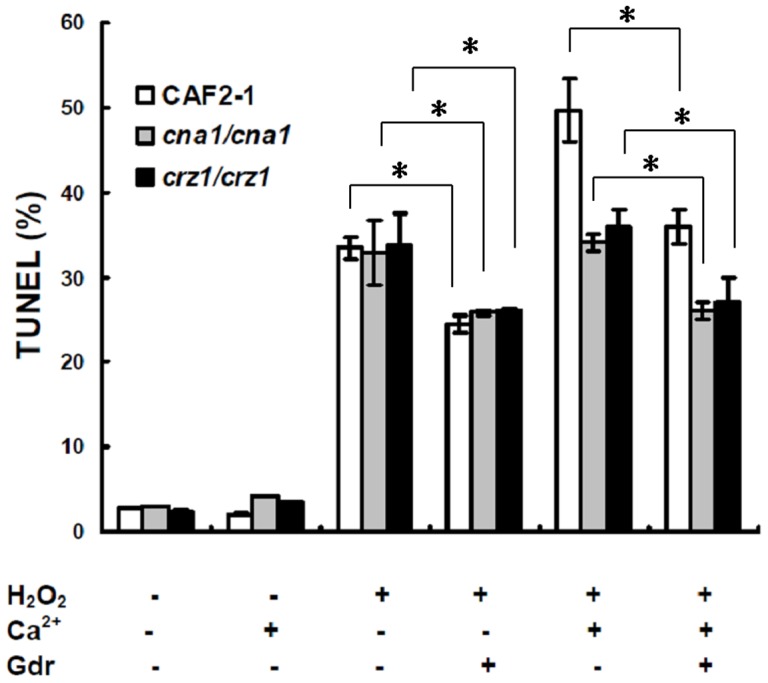
The impact of compromised Hsp90 on calcinerin-pathway-defect cells. *C. albicans* cells were treated with 1.25 mM H_2_O_2_ for 3 h in the absence or presence of CaCl_2_ (1 mM) or GdA (0.5 μM). Data are shown as mean ± SD from three independent experiments. * indicates p<0.05 compared to the same strain, same H_2_O_2_ and CaCl_2_ treatment, and without GdA treatment.

**Figure 10:**
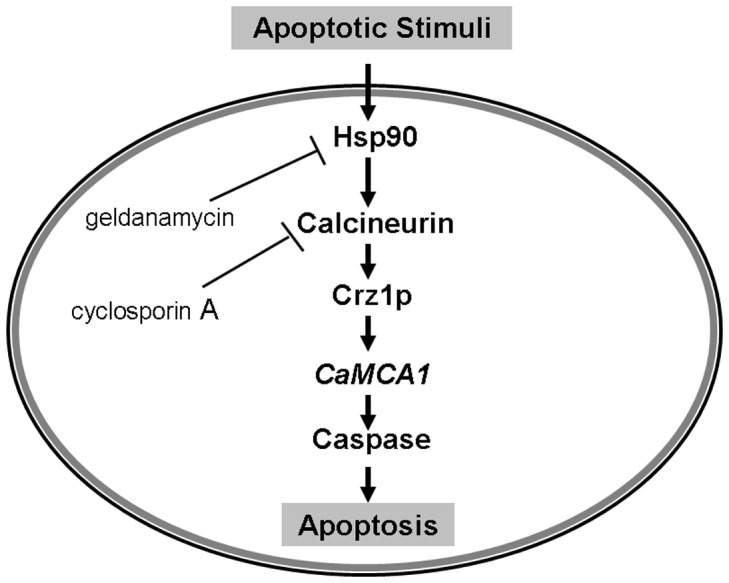
A model for the role of Hsp90 in regulating apoptosis in *C. albicans*. Upon apoptotic stimuli, Hsp90 was activated and *CaMCA1* expression was elevated. Elevation in *CaMCA1* expression resulted in increased caspase activity, thus inducing apoptosis. Calcineurin inhibitor cyclosporin A or Hsp90 inhibitor geldanamycin can block this pathway.

**Table 7:** *C. albicans* strains used in this study.

Strain	Parental strain	Genotype	Reference
HSP90/HSP90(SN95)	SC5314	*arg4Δ/arg4Δhis1Δ/his1Δ URA3/ura3::imm434*	Noble *et* *al*., 2005
tetO-HSP90/hsp90	SN95	*hsp90::CdHIS1/tetO-HSP90*	Singh *et* *al*., 2009
CAF2-1	SC5314	*ura3 Δ ::imm434/URA3*	Fonzi *et* *al.*, 1993
DSY2195	DSY2188	*crz1Δ::hisG/crz1Δ::hisG::URA3::hisG*	Karababa *et* *al.,* 2006
DSY2091	CAF4-2	*cnaΔ::hisG/cnaΔ::hisG::URA3::hisG*	Sanglard *et* *al*.,2003

Similar results were obtained from the caspase activity detection by using D_2_R staining. After 5 mM H_2_O_2_ treatment for 3 h in the presence of 20 μg/ml DOX, the percentage of wild-type cells stainable by D_2_R was 71.99%, while the percentage of tetO-HSP90/hsp90 cells was 35.42% (p<0.05, Table 4), indicating that caspase activity was decreased in tetO-HSP90/hsp90 cells. The results obtained from confocal laser microscopy confirmed the above result: the number of D_2_R staining positive cells from HSP90/HSP90 cells was significantly larger than that from *HSP90* depleted tetO-HSP90/hsp90 cells after the same treatment (Fig. 7).

Consistent results were obtained by using 60 mM AA (Table 5) and 2 μg/ml AMB (Table 6) as the apoptotic stimuli. After 60 mM AA treatment for 3 h with DOX, the percentage of D_2_R stainable cells was 42.43% in the wild-type strain, while it significantly decreased to 18.59% in the tetO-HSP90/hsp90 strain (p<0.05). After 2 μg/ml AMB treatment for 3 h by given DOX, the percentage of D_2_R stainable cells were 51.92% in the wild-type strain, while it significantly decreased to 22.83% in the tetO-HSP90/hsp90 strain (p<0.05), indicating that caspase activation was defective in *HSP90* depleted tetO-HSP90/hsp90 cells.

To further reveal the impact of Hsp90 on caspase activity, Hsp90 inhibitor geldanamycin was also exploited (Fig. 3C). As shown in Fig. 3C, addition of GdA significantly decreased the proportion of D_2_R stainable cells upon H_2_O_2_ treatment (P<0.05, Fig. 3C), which is consistent with the result obtained from genetic depletion of *HSP90*.

### The role of calcineurin in apoptosis of Hsp90 compromised cells

We reported in our previous study that calcineurin pathway was required for H_2_O_2_-induced *C. albicans* apoptosis through regulating *CaMCA1* expression and caspase activity. Our interest in this study was the relationship between calcineurin and Hsp90 in apoptosis. Based on previous publications [27], [28], [30], calcineurin was assumed to be downstream of Hsp90 and regulated by Hsp90. In this work, we confirmed that calcineurin was downstream of Hsp90 in apoptosis: activated calcineurin still up-regulated apoptosis in Hsp90-compromised cells, and inactivated calcineurin was unable to further down-regulate apoptosis in Hsp90-compromised cells. Our previous study found that cyclosporin A (CsA) at the concentration of 0.08 μM decrease apoptosis by inhibiting calcineurin [14], and therefore we used this concentration in this study. Briefly, compromising the calcineurin function by CsA administration significantly decreased apoptosis in wild-type HSP90/HSP90 strain cells (Fig. 8A), while it was unable to further decrease apoptosis in Hsp90-compromised strain cells (Fig. 8C). However, activating calcineurin function by giving 1mmol Ca^2+^ significantly increased apoptosis in both wild-type HSP90/HSP90 and Hsp90-compromised strain cells after 1.25 mM H_2_O_2_ treatment through TUNEL assay (Fig. 8A and Fig. 8C). The similar result was also obtained from caspase activity detection using D_2_R dye (Fig. 8B and Fig. 8D). These results indicate that calcineurin was downstream of Hsp90 in the apoptosis-regulating pathway.

### Compromising Hsp90 function has a stronger effect than compromising calcineurin function on apoptosis

To investigate the calcineurin function in Hsp90-mediated apoptosis, further experiments were carried out. Hsp90 inhibitor GdA was used on both wild-type strain (CAF2-1) and the calcineurin-pathway-defect strain, including *crz1Δ* mutant (DSY2195) and *cna1Δ* mutant (DSY2091). The calcineurin-pathway-defect genotype was confirmed because the addition of Ca^2+^ was unable to up-regulate apoptosis in DSY2195 and DSY2091 upon H_2_O_2_ treatment. Addition of Hsp90 inhibitor GdA also significantly decreased apoptosis of the two calcineurin-pathway-defect strains, DSY2195 and DSY2091 (P<0.05, Fig. 9) upon H_2_O_2_ treatment, indicating that compromising Hsp90 function had a stronger effect than compromising calcineurin function on apoptosis.

## Discussion

In recent years, the role of Hsp90 in apoptosis has been extensively investigated in mammalian cells [31]–[34]. It was reported that Hsp90 expression increased apoptosis in monoblastoid cell line U937 [31], while other studies [32] reported that Hsp90 played a protective role against 3-hydroxykynurenine-induced apoptosis in neurons. Further investigations in mammalian cells demonstrated that Hsp90 was involved in apoptosis through regulating caspase-9 and caspase-3 [33]. In contrast to mammalian cells, the role of Hsp90 in *C. albicans* apoptosis remained unclear before this study. In this work, we demonstrated that compromised Hsp90 reduced apoptosis in *C. albicans*. We first investigated the impact of Hsp90 compromise on apoptosis and cell fate. Upon application of the three different apoptotic stimuli used in this study, Hsp90-compromised tetO-HSP90/hsp90 cells tended to develop less apoptosis, less necrosis, longer survival and less ROS production compared with HSP90/HSP90 wild-type cells. Similar results were obtained by using the Hsp90 inhibitor GdA. Secondly, we investigated the impact of Hsp90 compromise on *CaMCA1* gene expression and caspase activity. Hsp90-compromised cells were defective in its ability to up-regulate *CaMCA1* gene expression and to activate caspase activity upon all the apoptotic stimulations, which are consistent with the previous results. Thirdly, we investigated the relationship between calcineurin and Hsp90. Previous studies [24]–[30] demonstrated that calcineurin was regulated by Hsp90 in echinocandin resistance. In this study, we confirmed that calcineurin was downstream of Hsp90 in causing apoptosis because activated calcineurin could still up-regulate apoptosis in Hsp90-compromised cells while inactivated calcineurin could not further down-regulate apoptosis in Hsp90-compromised cells. Finally, we found that Hsp90 inhibitor GdA could further decrease apoptosis in calcineurin-pathway-defect strains, indicating that compromising Hsp90 function had a stronger effect than compromising calcineurin function on apoptosis.

The reduced *C. albicans* apoptosis in Hsp90-compromised cells was linked to the defect of *CaMCA1* up-regulation and caspase activation. Caspase activation has been recognized as the most important process linked to apoptosis in mammalian cells [35]. Upon a death stimulus, the initiator caspases were activated, and then a second group of caspases were activated, leading to programmed cell death or apoptosis [36]–[40]. In yeasts, apoptosis is also associated with the activation of the caspase (or named metacaspase) [7]–[9]. The relationship between caspase activation and apoptosis in mammalian cells and in yeasts is consistent with our findings in this study.

Calcineurin was demonstrated to be regulated by Hsp90 in other biochemical processes [26]–[28]. Our findings confirmed the relationship between calcineurin and Hsp90 in apoptosis of *C. albicans*. In the presence of calcium ions, calcineurin can be activated, and regulate its target genes [15]–[17]. Here, we demonstrated that with calcium ions, the activated calcineurin could up-regulate apoptosis, while inactivated calcineurin could not further down-regulate apoptosis in Hsp90-compromised cells, indicating that calcineurin was downstream of Hsp90 in the apoptosis-regulating pathway (Fig. 10). Upon apoptotic stimuli, Hsp90 was activated and *CaMCA1* expression was elevated. Elevation in *CaMCA1* expression resulted in increased caspase activity, thus inducing apoptosis. Calcineurin inhibitor cyclosporin A or Hsp90 inhibitor geldanamycin could block this pathway.

Hsp90 inhibitor GdA could further decrease apoptosis in calcineurin-pathway-defect strains, including *crz1Δ* mutant and *cna1Δ* mutant, indicating that compromising Hsp90 function had a stronger effect than compromising calcineurin function on apoptosis. Compromising calcineurin function with cyclosporin A or compromising Hsp90 function (either with geldanamycin or genetically) has essentially identical effects on apoptosis. Consistent with this observation, inhibiting both pathways together has the same result as inhibiting either pathway alone. However, inhibition of Hsp90 has a stronger effect than genetic deletion of either *CNA1* or *CRZ1*. In their study of exploring roles of Hsp90 and calcineurin in echinocandin responses, Singh et al [30] found that genetic deletion of *CNA1* and *CRZ1* had weaker effects on echinocandin tolerance than genetic deletion of *CNB1* or treatment with cyclosporin A. Therefore, it is possible that combination of a *CNB1* mutation with Hsp90 inhibition would have the same phenotype as deletion of *CNB1* or Hsp90 inhibition. Therefore, further experiments are needed to investigate the underlying mechanism.

Hsp90 is known as an essential molecular chaperone that regulates the stability and function of a variety of proteins, many of which act as regulators of cellular signaling [24]–[26]. A recent proteomic and genomic study in yeast [41] revealed that 198 putative physical interactions and 451 putative genetic and chemical-genetic interactions were connected with Hsp90. Besides the calcineurin pathway, other pathways that are under the regulation of Hsp90 remain to be studied in future.

In summary, we have demonstrated that compromised Hsp90 reduced apoptosis in *C. albicans* partially through regulating the calcineurin-caspase apoptotic pathway. This work may open the door for the study of Hsp90 in apoptosis of *C. albicans*.

## Materials and Methods

### Strains and growth conditions

*C. albicans* strains used in this study are listed in Table 7. *C. albicans* cells were grown in yeast extract/peptone/dextrose (YPD) broth as described previously [7], [29], [30]. Briefly, cells were grown overnight in YPD at 30°C, diluted to OD_600_ of 0.2 with 20 μg/ml doxycycline as indicated, and grown overnight. Cells were then diluted to OD_600_ of 0.2 in the same conditions and grown to mid-log phase. For hydrogen peroxide, acetic acid or amphotericin B treatment, *C. albicans* cells were harvested and resuspended in the same conditions containing hydrogen peroxide, acetic acid or Amphotericin B.

### Drugs

Hydrogen peroxide (H_2_O_2_), acetic acid (AA), amphotericin B (AMB), Geldanamycin (GdA), Doxycycline (DOX), Calcium chloride (CaCl_2_) and cyclosporin A (CsA) are purchased from Sigma-Aldrich.

### Quantitative Real-time RT-PCR

RNA isolation, cDNA synthesis and real-time RT-PCR amplification were performed as described previously [6], [14]. Exponentially growing *C. albicans* cells were treated with H_2_O_2_ or sterile water as the control for 3 h before RNA isolation. Experiments were carried out using the Chromo 4 Real-Time PCR System (Bio-Rad, USA). SYBR Green I (Takara) was used to monitor the amplified products. Gene-specific primers were designed according to the manufacturer's protocol. Primers for *HSP90* were 5′-GGGAATCTAACGCTGGTGGTAA-′3 and 5′- TTCGGTTTCTGGAACTTCTTTT-3′; primers for *CaMCA1* were 5′-TATAATAGACCTTCTGGAC-3′ and 5′-TTGGTGGACGAGAATAATG -3′; and primers for 18S rRNA were 5′-TCTTTCTTGATTTTGTGGGTGG-3′ and 5′- TCGATAGTCCCTCTAAGAAGTG-3′. The *C*_Τ_ value of 18S rRNA was subtracted from that of the gene of interest to obtain a Δ*C*_Τ_ value. The Δ*C*_Τ_ value of an arbitrary calibrator (e.g. an untreated control group) was subtracted from the Δ*C*_Τ_ value for each sample to obtain a ΔΔ*C*_Τ_ value. The gene expression level relative to the calibrator was expressed as 2^−ΔΔ*C*Τ^. Triplicate independent experiments were conducted to generate a mean value.

### Apoptosis, necrosis and overall viability assays

*C. albicans* cells were grown in YPD to exponential phase and then treated with different apoptotic stimuli for 3 h. To investigate the occurrence of apoptosis, a terminal deoxynucleotidyltransferase-mediated dUTP-biotin nick end labeling (TUNEL) assay by In Situ Cell Death Detection Kit (Roche Applied Sciences, Mannheim, Germany) was performed according to the manufacturer's instructions [7], [8]. In Brief, *C. albicans* cells were washed twice in PBS and then fixed with 3.6% paraformaldehyde. Cell samples were stored at 4°C until required. Cells were rinsed twice with PBS and then incubated with permeabilization solution for 2 minutes on ice. The cells were rinsed in PBS and labeled, using a solution of the label and enzyme solutions from In Situ Cell Death Detection Kit, appropriate controls labeled only with the label solution. The cells were incubated for 1 h at 37°C in a humidified atmosphere in the dark, rinsed in PBS. The number of cells determined to be positive by the TUNEL assay was quantified using a BD FACSCalibur flow cytometer with excitation and emission wavelength settings at 488 and 520 nm, respectively. Necrosis were assessed by detecting propidium iodide (PI) uptake of the *C. albicans* cells, using PI at the concentration of 20 μg/ml. Overall viability was assessed by using clonogenic assays. Cells at 1×10^7^ per ml were diluted in series and plated in triplicate on YPD and then incubated at 30°C for 48 h. Statistical analyses were performed using ANOVA. P<0.05 was considered significant.

### Caspase activity determination

Caspase activity was determined by detecting D_2_R staining using CaspSCREEN^TM^ Flow Cytometric Apoptosis Detection Kit (BioVision, U.S.A.) [14]. The culture samples were incubated according to the manufacturer's assay instructions and analyzed using flow cytometry with excitation at 488 nm and emission at 530 nm respectively.

### Measurement of ROS

Intracellular levels of ROS were measured using DCFH-DA (Molecular Probes, U.S.A.) [6], [14]. Briefly, exponentially growing *C. albicans* cells were collected by centrifugation and washed three times with PBS. Subsequently, the cell samples were adjusted to 2×10^7^ cells/ml. After incubation with 20 μg/ml of DCFH-DA for 30 min at 30°C, the cells were exposed to apoptotic stimuli and incubated at 30°C with constant shaking (200 rpm). At specified interval, cell samples were observed with a Leica TCS sp2 confocal scanning laser microscope with excitation at 485 nm and emission at 520 nm. Alternatively 1 ml of cell suspension was harvested and 100 μl of the supernatant was transferred to the wells of a flat-bottom microplate (BMG Microplate, 96well, Blank) to detect fluorescence intensities on the POLARstar Galaxy (BMG, Labtech, Offenburg, Germany) with excitation at 485 nm and emission at 520 nm. Each experiment was performed in triplicate.

### Statistical analysis

All experiments were performed in triplicate and at least three independent experiments were done on separate occasions. Data are presented as mean ± SD, and the ANOVA test was employed to determine the statistical significance between experimental groups. The difference was considered significant if the *P* value was less than 0.05.
